# Coordination of the dynamics of yeast sphingolipid metabolism during the diauxic shift

**DOI:** 10.1186/1742-4682-4-42

**Published:** 2007-10-31

**Authors:** Fernando Alvarez-Vasquez, Kellie J Sims, Eberhard O Voit, Yusuf A Hannun

**Affiliations:** 1 Dept. of Biostatistics, Bioinformatics and Epidemiology. Medical University of South Carolina, Charleston, SC. USA; 2 Dept. of Biochemistry and Molecular Biology. Medical University of South Carolina, Charleston, SC. USA; 3 Wallace H. Coulter Dept. of Biomedical Engineering. Georgia Institute of Technology, Atlanta, GA USA

## Abstract

**Background:**

The diauxic shift in yeast requires cells to coordinate a complicated response that involves numerous genes and metabolic processes. It is unknown whether responses of this type are mediated *in vivo *through changes in a few "key" genes and enzymes, which are mathematically characterized by high sensitivities, or whether they are based on many small changes in genes and enzymes that are not particularly sensitive. In contrast to global assessments of changes in gene or protein interaction networks, we study here control aspects of the diauxic shift by performing a detailed analysis of one specific pathway–sphingolipid metabolism–which is known to have signaling functions and is associated with a wide variety of stress responses.

**Results:**

The approach uses two components: publicly available sets of expression data of sphingolipid genes and a recently developed Generalized Mass Action (GMA) mathematical model of the sphingolipid pathway. In one line of exploration, we analyze the sensitivity of the model with respect to enzyme activities, and thus gene expression. Complementary to this approach, we convert the gene expression data into changes in enzyme activities and then predict metabolic consequences by means of the mathematical model. It was found that most of the sensitivities in the model are low in magnitude, but that some stand out as relatively high. This information was then deployed to test whether the cell uses a few of the very sensitive pathway steps to mount a response or whether the control is distributed throughout the pathway. Pilot experiments confirm qualitatively and in part quantitatively the predictions of a group of metabolite simulations.

**Conclusion:**

The results indicate that yeast coordinates sphingolipid mediated changes during the diauxic shift through an array of small changes in many genes and enzymes, rather than relying on a strategy involving a few select genes with high sensitivity. This study also highlights a novel approach in coupling data mining with mathematical modeling in order to evaluate specific metabolic pathways.

## 1. Introduction

Yeast cells challenged by depletion of their preferred carbon sources in the surrounding medium begin using other available carbons for energy production. This switch, usually from glucose to ethanol and acetate, is known as the *diauxic shift*. It is not surprising that the diauxic shift constitutes a very complicated dynamic process that requires fine tuned coordination at the genomic and biochemical levels. At the genomic level, the switch to secondary non-fermentable carbon sources necessitates sweeping changes in gene regulation, which have been assessed with microarrays measured at a series of time points [1,2]

Specifically about the time of diauxic shift, the cells begin up-regulating hundreds of genes, which are associated with respiration, fatty acid metabolism and the launch of an environmental stress response, while down-regulating other genes whose products are no longer needed in prior amounts (*e.g*., [3]). In turn, at the biochemical level, these changes in gene expression lead to altered metabolic, enzymatic, and flux profiles. Connecting the two levels are mechanisms of signal transduction that respond to the depletion of primary substrate and ultimately effect genomic adjustments.

As such, published microarray data contain a hidden wealth of information, and often specific aspects of cell regulation are of interest to particular investigators. Therefore, there are increasing needs to develop approaches that allow extraction of relevant data and then applying specific analytical methods on these data in order to predict functional consequences. In this study, we focus on sphingolipid metabolism and changes that occur during the diauxic shift. The choice of this pathway system was based on the fact that sphingolipids have been recognized in yeast and other eukaryotes as important signaling molecules that respond to a variety of stresses and are crucially involved in the coordination of stress responses [4]. The overall strategy of this work is to translate published information on changes in gene expression during the diauxic shift into alterations in enzyme activities and to deduce, by means of a mathematical model, subsequent changes in metabolic profiles within the sphingolipid pathway.

In a pilot study using a similar strategy, we previously translated global mRNA microarray results into a mathematical pathway model, which was then employed to study the coordination of the glycolytic pathway in *Saccharomyces cerevisiae *following the initiation of heat stress [5]. Using similar mathematical arguments, we investigated the coordination of regulation in the trehalose cycle [6]. Analyzing heat shock in a slightly different fashion, Vilaprinyo [7] used microarray data for testing evolutionary implications of changes in gene expression. Adapting the methodologies of these earlier studies, we are here importing results from microarray time series during the diauxic shift [1,2] into a mathematical model with the goal of characterizing dynamic changes in the sphingolipid pathway at the metabolic and physiologic levels.

The two published microarray data on the diauxic shift consist of global mRNA measurements at seven time points, spanning a period of about 12 hours [1] and 11 hours [2], respectively, during which the yeast culture switched from glucose fermentation to respiration of ethanol and acetate and the production of large amounts of ATP.

Specifically, we are interested in changes within the (sphingo)lipidomic profile between a baseline fermentative state during exponential growth (at 11 hours of batch culturing) and a later time point at 21 hours, which corresponds to respiration after the diauxic shift [1]. At this time, glucose is depleted, but the cell density is still increasing, though with decreased growth rate, and the cell culture has not reached stationary state. During this phase, cell growth and division continue to require lipid production for inclusion in the membrane of internal organelles and the plasma membrane.

**Figure 1:**
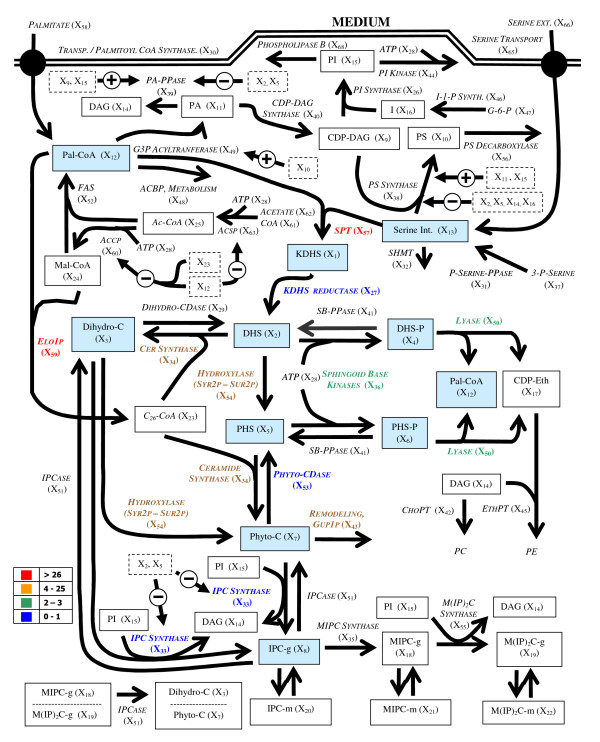
Sphingolipid-glycerolipid model for yeast. Solid boxes represent time dependent variables, italics represent variables assumed to be constant (time independent), dashed boxes represent variables with inhibitory or activating effects. Blue boxes represent metabolite log gains analyzed in this work. The color scale corresponds to the summed absolute values of metabolite log gains for the enzymes of the sphingolipid block listed in Table 2 (see text for details).

Complementing the microarray data [1,2], our analysis makes use of a variety of biochemical, regulatory and genetic pieces of information on the sphingolipid pathway. This information was recently collated and integrated into a comprehensive kinetic-dynamic mathematical model [8] and is represented in Fig. 1. The model was thoroughly diagnosed and subsequently subjected to experimental validation [9].

An important component of a typical model assessment is the analysis of its sensitivity to changes in parameters and independent variables. The former may be *K*_*M *_values in Michaelis-Menten models or rate constants and kinetic orders in power-law models, while the latter typically refer to enzyme activities and input variables, such as substrates and other precursors or modulators. Relative changes in model output that are caused by small perturbations in independent variables are called *logarithmic gains *(Log Gains; LG; [10]). These LG can serve both as diagnostic and predictive tools accompanying the model. If the gains are small in magnitude, perturbations are rather inconsequential. By contrast, large gains indicate that the system responds strongly to changes in a given independent variable. A strong response may be advantageous or not. On one hand, the system should be robust to naturally occurring random fluctuations in conditions, which would mandate gains of small size. On the other hand, signal transduction systems must react strongly to relevant inputs and amplify them multifold to evoke an appropriate response.

Sphingolipid metabolism constitutes an interesting system, as it is biochemical in nature and should therefore be robust, exhibiting small gains. At the same time, some of the sphingolipids and their relative amounts serve as signaling molecules, which therefore have to respond forcefully to the sensing of specific, and often adverse, environmental conditions such as heat shock or oxidative stress. For these reasons of contrasting demands, it is interesting to study log gain profiles of the sphingolipid pathway in detail. We execute this analysis here, focusing on functional clusters of variables and fluxes of primary significance, and compare our findings to results characterizing diauxic shift conditions. Given the complexity of the pathway one should expect that there are multiple ways of genomic and metabolic switching from the pre-diauxic metabolic profile to one that is suited for post-diauxic conditions. To gain insight into this switch, we will study the specific question of whether yeast employs a few independent variables (enzymes) with high log gains that are able to effect appropriate changes in metabolic profile during diauxic shift, or whether larger numbers of enzymes are adjusted only slightly. We will also explore whether there is a preference for exerting control through changes in precursors or in enzyme activities.

Finally, we discuss the utility of this approach as a prototype that can be employed towards 'mining' pathway-specific data from the ever-increasing numbers of published microarrays, and then using these data to predict functional metabolic consequences.

## 2. Methods

The analysis is overall divided in three parts, which are all executed with a recent mathematical model of sphingolipid metabolism (Fig. 1; [8,9]). The model, along with slight modifications accounting for new experimental findings, is discussed in Section 2.1 and the Appendix. Section 2.2 describes the computation of sphingolipid related logarithmic gains, and Section 2.3 discusses our implementation of processes associated with the diauxic shift characterized in the published microarray expression data. Most of the analyses were executed with PLAS [11] and MAPLE [12].

### 2.1. – Specific modifications to the model

The model was taken essentially as described in our earlier work [8,9]. One exception is internal serine, which we considered constant in the present model. This change appeared reasonable because new experiments have shown that its measured internal value is maintained at a very stable concentration during the diauxic shift (Cowart A., personal communication). Furthermore, serine is not only a starting metabolite for the glycerolipid and sphingolipid pathways but also participates in other metabolic routes that are not represented in the model, such as the folate cycle, as well as protein synthesis (*e.g*., [13,14]). Since these paths of serine utilization are not modeled, perturbations would lead to undue accumulation in the model. A few other minor modifications to the model are described in the Appendix.

### 2.2. – Logarithmic Gains: Measurements of the Sensitivity of the Model

One of the most widely used quantitative criteria of model quality and robustness is parameter sensitivity.

In a comprehensive sensitivity analysis, each parameter is modulated by a small amount, and the effects of this modulation on steady-state concentrations and fluxes (*e.g*., [10,15]), or on transients (*e.g*., [16,17]) are analyzed. The analysis is typically executed through partial differentiation at a chosen operating point.

Among various types of sensitivities, analyses of so-called *logarithmic gains *(LG), which have been successfully applied to moderately large biological systems (*e.g*., [18-20]), are of particular importance here. An LG quantifies the effect that a small (strictly speaking, infinitesimal) perturbation in a given independent variable has on the steady-state values of metabolite concentrations or fluxes in the system. Mathematical details are presented in the Appendix.

An LG with magnitude greater than 1 implies amplification of the perturbation; thus, a 1% change in the independent variable evokes more than 1% in the steady-state output quantity. A magnitude less than 1 indicates attenuation. A positive sign for the LG indicates that the changes are in the same direction, so that both increase in value or both decrease. A negative sign indicates that the changes are in opposite directions.

In typical, robust models of metabolic pathways, the majority of LGs are in a range between -1 and 1, which indicates that perturbations in most independent variables are attenuated by the system. LGs with a magnitude between 1 and 5 characterize the effect of moderate amplification. LGs of much higher magnitude typically have one of three causes. The particular independent variable may truly have a high gain, which is, for instance, the case in signaling systems whose role it is to amplify weak incoming signals. Second, the independent or the dependent variable associated with the LG is at the fringes of the model, and the high gain is an artifact due to processes that in reality contribute to the dynamics (*e.g*., further metabolism) of this variable but are not included in the model. These additional processes tend to buffer the variable against perturbations. Third, a variable associated with a high LG is not modeled with sufficient accuracy. It could be that a very inaccurate value is assigned to a parameter or that some production or degradation processes are missing. True high gains are interesting because they allow the cell to effect a desired change or adaptation to a new situation with relatively modest effort. At the same time, high gains are obviously difficult to control. We will analyze in a later section to what degree yeast may employ high-gain variables to organize the diauxic shift from fermentation to respiration.

One should note that each LG addresses the perturbation in one independent variable and its impact on one dependent variable at a time. The effects of multiple simultaneous perturbations can in principle be assessed with a "synergism analysis" [21,22], which however is mathematically very involved even in the simplest cases of two combined changes, where tensor analysis replaces the simple matrix computations of LG analysis. An alternative is a comprehensive computational analysis, where multiple, finite, perturbations are introduced in the model and the effects are studied. In the system under consideration here, 34 enzymes would need to be considered, leading to more than 1,000 pair-wise analyses for each of the twenty-five dependent variables, if positive and negative perturbations were to be tested. For triplet perturbations, the number per dependent variable would jump to about 12,000. Because we use LG primarily as indicators of relative importance, we do not pursue synergism analyses here.

In the current model there are twenty-five dependent and forty independent variables, so that the complete analysis just with respect to metabolite LG involves more than 2,000 quantities, most of which are close to 0 and not particularly interesting.

For the current analysis, we focused on LGs for metabolites and fluxes of the sphingolipid core, *i.e*., 3-keto-dihydrosphingosine (KDHS, *X*_1_), dihydrosphingosine (DHS, *X*_2_), dihydroceramide (Dihydro-C, *X*_3_), dihydrosphingosine-1P (DHS-P, *X*_4_), phytosphingosine (PHS, *X*_5_), phytosphingosine-1P (PHS-P, *X*_6_), phytoceramide (Phyto-C, *X*_7_), inositol phosphorylceramide (IPC-g, *X*_8_), palmitoyl-CoA (Pal-CoA, *X*_12_), and serine (Serine Int., *X*_13_). They are represented in the diagram of Fig. 1 as boxes shaded in blue and listed in Table 1. Furthermore, in a new variation on this type of analysis, we studied the effects on functional blocks of output quantities instead of individual outputs. Specifically, we dissected the pathway in three blocks: metabolic pathways precursors, sphingolipids, and glycerolipids.

**Table 1:** Steady-state metabolite levels corresponding to mRNA profiles

			**FOLD CHANGE (normalized against 11 hr)**						
			
**Abbreviation**	**Symbol**	**Value (mol%)**	**9 hr**	**11 hr**	**13 hr**	**15 hr**	**17 hr**	**19 hr**	**21 hr**
KDHS	*X*_1_	0.005	0.98	1	0.78	1.01	1.27	0.80	1.55
DHS	*X*_2_	0.01	1.13	1	0.53	0.77	0.50	1.98	7.88
Dihydro-C	*X*_3_	0.036	1.65	1	0.76	2.07	1.24	0.95	4.28
DHS-P	*X*_4_	0.001	1.27	1	0.36	0.48	0.32	2.31	15.76
PHS	*X*_5_	0.05	0.89	1	0.43	0.54	0.34	3.00	4.73
PHS-P	*X*_6_	0.005	0.99	1	0.24	0.29	0.18	4.21	12.96
Phyto-C	*X*_7_	0.052	0.86	1	0.51	0.51	0.55	0.33	1.02
IPC-g	*X*_8_	0.102	1.93	1	0.06	0.61	0.80	0.003	3.71
CDP-DAG	*X*_9_	5.4	1.18	1	0.44	0.72	0.95	0.76	3.13
PS	*X*_10_	8.4	0.77	1	1.22	2.79	3.37	4.15	8.14
PA	*X*_11_	3	1.06	1	0.82	1.51	2.11	2.46	5.11
Pal-CoA	*X*_12_	0.01 ^(*)^	0.97	1	0.96	1.17	1.14	0.95	1.29
Serine	*X*_13_	2600 ^(*)^	1	1	1	1	1	1	1
DAG, X14	*X*_14_	0.1	1.25	1	0.97	1.18	1.80	1.83	3.94
PI, X15	*X*_15_	16.7	1.03	1	0.57	0.78	1.14	0.36	1.74
Inositol	*X*_16_	24.1 ^(*)^	1	1	1	1	1	1	1
CDP-Eth	*X*_17_	22	0.55	1	0.03	0.04	0.01	3.20	15.25
MIPC-g	*X*_18_	0.14	1.56	1	0.37	1.25	1.17	0.07	2.02
M(IP)2C-g	*X*_19_	0.0085	1.47	1	0.25	0.77	1.43	0.05	3.31
IPC-m	*X*_20_	0.918	1.93	1	0.06	0.61	0.80	0.003	3.71
MIPC-m	*X*_21_	1.26	1.56	1	0.37	1.25	1.17	0.07	2.02
M(IP)2C-m	*X*_22_	0.0765	1.47	1	0.25	0.77	1.43	0.05	3.31
C26-CoA	*X*_23_	0.5	0.79	1	4.33	1.97	4.21	0.47	0.06
Mal-CoA	*X*_24_	183 ^(*)^	1.04	1	0.35	0.46	0.31	1.20	11.60
Ac-CoA	*X*_25_	870 ^(*)^	1	1	1	1	1	1	1
Total IPC	*X*_8 _+ *X*_20_	1.02	1.93	1	0.06	0.61	0.80	0.003	3.71
Total MIPC	*X*_18 _+ *X*_21_	1.4	1.56	1	0.37	1.25	1.17	0.07	2.02
Total MIP_2_C	*X*_19 _+ *X*_22_	0.085	1.47	1	0.25	0.77	1.43	0.05	3.31
Total_Ceramide	*X*_3 _+ *X*_7_	0.088	1.18	1	0.61	1.15	0.83	0.59	2.35

(*) μM. Steady-state metabolite levels corresponding to microarray time course data during the diauxic shift (from DeRisi *et al*. [1]). Each case is represented as fold change of the value presented in Alvarez-Vasquez *et al*. [9]

### 2.3. – Strategy for Implementing Dynamic Changes during Diauxic Shift

The LG analysis described above characterizes the robustness of the model with respect to a given, small perturbation. In contrast to such small alterations in values, a coordinated cellular response such as the diauxic shift from fermentation to respiration is associated with multiple changes in gene expression and enzyme activities, which are not necessarily small. To analyze this response, we used two sets of published time series of yeast microarray data [1,2] one for the primary analysis [1] and the second for evaluating the reproducibility of the metabolomic output [2].

**Figure 2:**
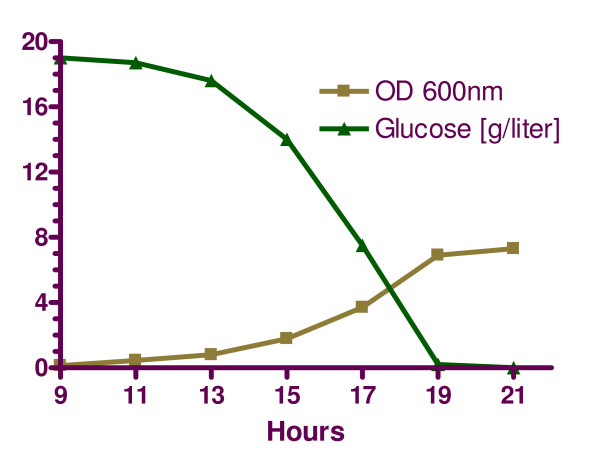
Cellular density and external glucose concentration during the time period when genomic expression was measured. mRNA levels at 11 hrs are assumed to correspond to the model of [9]. Adapted from DeRisi ([1], Fig 5A).

DeRisi *et al*. [1] quantified changes in yeast gene expression with microarray experiments that were spaced in two-hour intervals from 9 hrs to 21 hrs of batch culture. Measurements were done with a wild type strain growing in YPD medium at 30°C, and the study also reported the levels of glucose and cellular densities at the experimental time points (Fig. 2). To ensure maximal consistency with the model, we chose the 11-hour time point as baseline, because it falls within the exponential growth phase for which the model parameters were originally selected. Since DeRisi's experimental results consist of ratios of mRNA expression over baseline, all expression levels were divided by the 11-hour levels, so that the 11-hour measurements became "normal" levels of 1 unit. Table 2 shows the enzymatic specific activities in the model at the 11-hour reference point. In the sphingolipid model, several steps are catalyzed with isozymes, such as the sphingoid base kinase (*X*_36_), phosphatidate phosphatase (*X*_39_), G3P acyltransferase (*X*_49_), and ELO1p (*X*_59_), or by different subunits such as FAS (*X*_52_) and SPT (*X*_57_). The contributions of these isozymes and subunits were weighted against their corresponding mRNA isoenzymes or subunits.

**Table 2:** Specific enzyme activities

	**Abbreviation**		**Activity (U/mg)**	**Ref.**
**GLYCEROLIPID BLOCK:**				
Phosphatidylinositol Synthase	PI Synthase	*X*_26_	0.00266	[57]
Phosphatidylserine Synthase	PS Synthase	*X*_38_	0.00332	[57]
Phosphatidate Phosphatase	PA-Ppase	*X*_39_	0.0024	[58]
CDP-Diacylglycerol Synthase	CDP-DAG Synthase	*X*_40_	0.00061	[59]
DG-Choline Phosphotransferase	ChoPT	*X*_42_	0.00066	[60]
Phosphoinositide Kinase	PI Kinase	*X*_44_	0.00172	[61]
Diacylglycerol-Ethanolamine Phosphotransferase	EthPT	*X*_45_	0.001	[60]
Inositol-1-P Synthase	I-1-P Synth	*X*_46_	0.000833	[62]
Glycerol-3-Phosphate Acyltransferase	G3P Acyltranferase	*X*_49_	0.00394	[63]
Phosphatidylserine Decarboxilase	PS Decarboxylase	*X*_56_	0.00001066	[64]
Phospholipase B	Phospholipase B	*X*_68_	0.0005	δ
**SPHINGOLIPID BLOCK:**				
3-Ketodihydrosphingosine Reductase	KDHS Reductase	*X*_27_	0.000262	[65]
Dihydroceramide Alkaline Ceramidase	Dihydro-Cdase	*X*_29_	0.0000054	[66]
Inositol Phosphorylceramide Synthase	IPC Synthase	*X*_33_	0.00033	[67]
Ceramide Synthase	Cer Synthase	*X*_34_	0.0000165	[68]
Mannosyl Inositol Phosphoceramide Synthase	MIPC Synthase	*X*_35_	0.000165	[8, 69]
Sphingoid Base Kinase	Sphingoid Base Kinase	*X*_36_	0.000004	[43]
Sphingoid-1-phosphate Phosphatase	SB-Ppase	*X*_41_	0.0008	[70]
GUP1p	GUP1p	*X*_43_	0.0001	δ
Sphingosine-Phosphate Lyase	Lyase	*X*_50_	0.0000367	[71]
IPCase, Phyto-C formation	IPCase	*X*_51_	0.00015	[72]
Phytoceramide Ceramidase	Phyto-Cdase	*X*_53_	0.0000198	[66]
4-Hydroxylase	Hydroxylase	*X*_54_	0.00017	
Mannosyldiinositol Phosphorylceramide Synthase	M(IP)2C Synthase	*X*_55_	0.0000825	[8, 69]
Serine Palmitoyltransferase	SPT	*X*_57_	0.000106	[65]
Very Long Chain Fatty Acid Synthase	ELO1p	*X*_59_	0.0006	[73]
IPCase, Dihydro-C formation	IPCase	*X*_64_	0.00015	[72]
**PRECURSOR BLOCK:**				
Transport/Palmitoyl CoA Synthase	Transp./Palmitoyl CoA Synthase	X_30_	0.0508	[74]
Phosphoserine-Phosphatase	P-Serine-PPase	*X*_31_	0.0013	[75]
Serine Hydroxymethyl Transferase	SHMT	*X*_32_	0.0045	[76]
Acyl-CoA-Binding Protein	ACBP	*X*_48_	20 ^(*)^	[77]
Fatty Acid Synthetase	FAS	*X*_52_	0.0089	[78]
Acetyl-Coenzyme A Carboxylase	ACCp	*X*_60_	0.022	[79]
Acetyl-Coenzyme A Synthetase	ACSp	*X*_63_	0.73	[80]
Serine Transport	Serine Transport	*X*_65_	0.0193224	[81, 82]

(*) μM. (δ) Estimated. Specific enzyme activities during the exponential growth phase; from Alvarez-Vasquez *et al*. [9]. The enzymes were categorized into glycerolipid, sphingolipid, and precursor blocks.

The ACSp (*X*_63_) isoenzymes were not weighted because their product (Ac-CoA) is considered an independent variable in the model.

For example, at 9 hrs, the two reported isoenzymes for phosphatidate phosphatase (*X*_39_), represented in Table 3, were weighted against their corresponding highest normalized mRNA values as:

**Table 3:** Fold changes in mRNA's of model enzymes

**Gene**	**ORF's**	**9 hr**	**11 hr**	**13 hr**	**15 hr**	**17 hr**	**19 hr**	**21 hr**
		**Fold Increases in mRNA (DeRisi *et al *., 1997)**						
		
*LCB4*	YOR171C	1.1	1.16	1.3	0.92	1.02	1.61	1.37
*LCB5*	YLR260W	0.88	0.81	0.97	0.9	0.77	2.13	1.35
								
*DPP1*	YDR284C	0.93	0.91	1.67	1.15	0.98	1.54	0.83
*LPP1*	YDR503C	1.08	1.04	1.47	1.05	1.15	1.18	1.27
								
*GPT2*	YKR067W	1.01	1.05	1.16	1.35	1.64	2.10	2.36
*GAT2/SCT1*	YBL011W	0.96	0.94	1.09	0.83	0.85	1.15	0.72
								
*FAS1*	YKL182W	1.11	1.08	0.97	0.83	0.78	0.88	0.77
*FAS2*	YPL231W	0.95	1.03	0.95	0.92	0.71	0.71	0.71
								
*LCB1*	YMR296C	0.97	1	1.16	0.82	0.90	1.04	1.19
*LCB2/SCS1*	YDR062W	0.96	1.04	1.14	0.93	0.83	0.45	0.40
								
*ELO1*	YJL196C	0.95	0.85	1.23	1.09	1.33	0.79	0.41
*ELO2/FEN1*	YCR034W	1.09	1.11	1.89	1.01	0.75	0.54	0.32
*ELO3/SUR4*	YLR372W	1.06	1.27	1.56	0.95	1.05	0.43	0.23
		**Fold Increases normalized against 11 hr values**						
		
*LCB4*	YOR171C	0.94	1	1.11	0.79	0.88	1.40	1.19
*LCB5*	YLR260W	1.08	1	1.20	1.11	0.95	2.65	1.66
								
*DPP1*	YDR284C	1.02	1	1.84	1.26	1.08	1.69	0.91
*LPP1*	YDR503C	1.03	1	1.41	1.01	1.10	1.13	1.22
								
*GPT2*	YKR067W	0.97	1	1.11	1.29	1.57	2.00	2.25
*GAT2/SCT1*	YBL011W	1.03	1	1.15	0.88	0.90	1.22	0.77
								
*FAS1*	YKL182W	1.04	1	0.90	0.77	0.72	0.82	0.72
*FAS2*	YPL231W	0.93	1	0.93	0.89	0.69	0.69	0.70
								
*LCB1*	YMR296C	0.98	1	1.16	0.82	0.91	1.05	1.20
*LCB2/SCS1*	YDR062W	0.93	1	1.09	0.90	0.80	0.44	0.39
								
*ELO1*	YJL196C	1.11	1	1.45	1.27	1.57	0.92	0.48
*ELO2/FEN1*	YCR034W	0.99	1	1.69	0.91	0.67	0.49	0.29
*ELO3/SUR4*	YLR372W	0.84	1	1.22	0.75	0.83	0.34	0.18

mRNA fold changes and corresponding values of enzyme activities in the model at the different time point, normalized against 11 hr data. Data from DeRisi *et al*. [1]. *LCB4 *– *LCB5 *and *DPP1-LPP1 *are a pair of enzymes with similar substrates and/or products and they represent the sphingoid base kinase (*X*_36_) and the phosphatidate phosphatase (*X*_39_), respectively. *FAS1- FAS2 *and *LCB1-LCB2 *correspond to sub-units for fatty acid synthase (*X*_52_) and serine palmitoyl transferase (*X*_57_), respectively. The three ELO's represent the battery of enzymes involved in fatty acid elongation represented in the model by Elo1p (*X*_59_).

mRNA_39 _= (1.02 × 1.02/1.84 + 1.03 × 1.03/1.41)/(1.02/1.84 + 1.03/1.41) = 1.03.

As a second example, the weighted phosphatidate phosphatase mRNA fold change at 19 hrs is computed as

mRNA_39 _= (1.69 × 1.69/1.84 + 1.13 × 1.13/1.41)/(1.69/1.84 + 1.13/1.41) = 1.43.

In a more refined analyses, one could represent each isozyme separately, which however would require more input data for model design.

### 2.4. – Validation Experiments

#### Yeast strain and growth conditions

Background strain BY4742 (MATα his3Δ1 leu2Δ0 lys2Δ0 ura3Δ0) from the yeast deletion library was first grown in an overnight culture of YPD from a freshly streaked plate of the frozen stock. Flasks of SC medium were then inoculated to a starting OD_600 _≅ 0.1 and incubated at 30°C and 220 rpm. Samples were taken after 6 hours (OD_600 _= 0.34) and 24 hours (OD_600 _= 2.2), spun down at 3000 rpm for 5 minutes, the supernatant removed, and the remaining cell pellet frozen at -80°C until lipid analysis.

#### Lipid extraction and measurement by mass spectrometry

Samples were fortified with internal standards, extracted with a solvent system modified from Mandala *et al*. [23] and then injected. ESI/MS/MS analysis was performed on a Thermo Finnigan TSQ 7000 triple quadrupole mass spectrometer, operating in a Multiple Reaction Monitoring (MRM) positive ionization mode [24]. Peaks corresponding to the target analytes and internal standards were collected and processed using the Xcalibur software system.

Quantitative analysis was based on the calibration curves generated by spiking an artificial matrix with the known amounts of the target analyte synthetic standards and an equal amount of the internal standards (ISs). The target analyte/IS peak areas ratios were plotted against analyte concentration. The target analyte/IS peak area ratios from the samples were similarly normalized to their respective ISs and compared to the calibration curves, using a linear regression model.

#### Sample normalization by lipid phosphates

The lipid concentrations from the mass spectrometry analysis were normalized by total lipid phosphate as determined with a standard curve analysis and colorimetric assay of ashed phosphate: aliquots of extracted samples were re-extracted via Bligh and Dyer [25] to separate the lipid-containing organic phase which was dried and assayed for phosphate content by ashing as previously described by Jenkins and Hannun in [26].

## 3. – Results

### 3.1. – Log Gains

Initially, the most relevant metabolites were grouped in three functional blocks and analyzed with respect to the flux and metabolite LG within each block. The blocks were chosen as: a) precursor block, including fatty acid metabolism and serine metabolism; b) sphingolipid block, including complex and backbone sphingolipids, which are crucial in cell regulation [27,28], and c) glycerolipids.

In Fig. 1, the LG associated with the sphingolipid block are colored according to their summed absolute values, ranking from highest to lowest impact by red, yellow, green, and blue.

**Figure 3:**
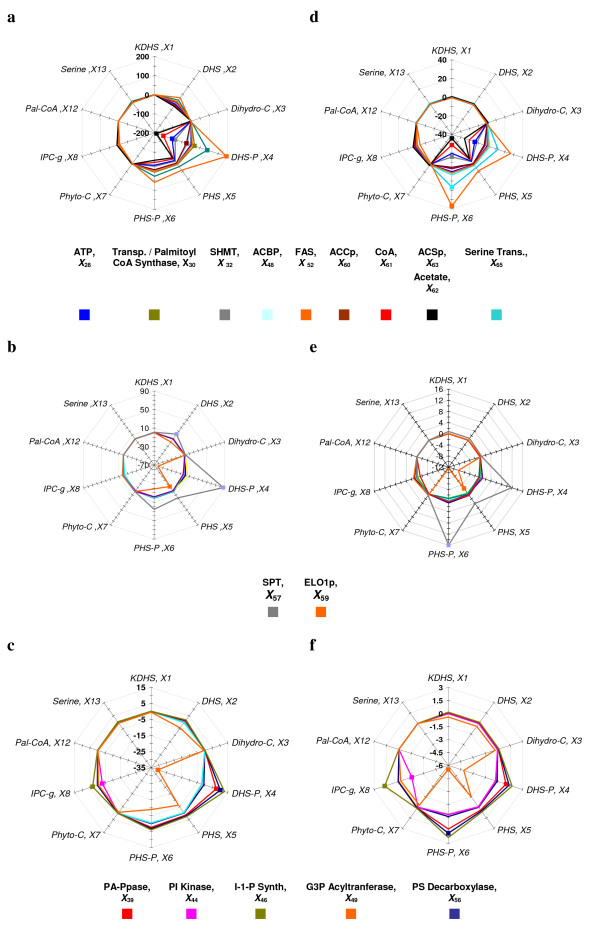
"Spider-web" representation of log gains in the model of Fig. 1 at the 11 hr time point. Log gains are summed for ten representative sphingolipid related metabolites or fluxes with respect to the time independent variable blocks listed in Table 2. Overlapping lines correspond to log gains with similar values. **a**. Metabolite Log Gains of the Precursor block. **b**. Metabolite Log Gains of the Sphingolipid block. **c**. Metabolite Log Gains of the Glycerolipid block. **d**. Flux Log Gains of the Precursor block. **e**. Flux Log Gains of the Sphingolipid block. **f**. Flux Log Gains of the Glycerolipid block.

In Fig. 3, the metabolite and flux LG are shown in a "spider-web" representation. In this representation, spikes to the outside of 1 exhibit magnification with direct proportionality, whereas spikes to the inside indicate that increases in the independent variable lead to decreases in the output variable. As an example, consider the system response in DHS-P (*X*_4_) to perturbations in independent variables, as shown in Fig. 3f. Most independent variables in this block have only a modest effect. This is seen by starting at the 12 o'clock spoke and following the polygon labeled 0 clockwise to the spoke labeled DHS-P, *X*_4_. The dark and light blue lines indicate that alterations in PS synthase and PI kinase activities have essentially no effect on the steady-state value of DHS-P. Following the spoke inward, one can see that a 1% increase in G3P acyltansferase leads to a steady-state DHS-P value that is decreased by about 4%. Thus, the LG is about -4. Looking at the corresponding spoke in Fig. 3e, a 1% increase in SPT is predicted to lead to an 11% increase in DHS-P.

The widest metabolite LG range was obtained inside the precursor block (+200,-200) followed by the sphingolipid block (+90,-70), and lastly by the glycerolipid block (+15, -35). The fluxes in all three blocks have smaller LG values than the metabolites, which means that the metabolic profile is more sensitive than the overall flux pattern.

#### 3.1.1. – Precursor Block

The LG pattern in this block is extreme (Figs. 3a,d). Some key variables, such as dihydroceramide and palmitoyl-CoA are essentially unaffected by any change in precursors. By contrast, DHS-P and PHS-P exhibit enormous sensitivity, followed by strong effects on DHS and PHS. The highest LGs by far are associated with the dynamics of Pal-CoA (*X*_12_) and Ac-CoA (*X*_25_), followed by serine (*X*_13_). Also high are LG for ACSp (*X*_63_) and FAS (*X*_52_), which is consistent with the crucial biological importance of these two enzymes for yeast viability: indeed, *ACS1/2 *double null mutant yeast strains and *FAS3 *knockout have been reported as non-viable [29,30].

The LG for Ac-CoA precursors have mostly an inverse effect on the sphingoid phosphates, which suggests that even small increases in Ac-CoA could lead to significant decreases in these metabolites. While the importance of Ac-CoA for sphingolipid dynamics is clear from this LG profile, the specific numerical values of the LG associated with Ac-CoA should at this point be considered merely as a measure of tendency. First, a 1% increase in Ac-CoA corresponds to an available Ac-CoA concentration of 8.7 μM of material into the system. This amount is very large in comparison to the normal sphingolipid concentrations. Second, it is known that Ac-CoA is involved in many processes that are not modeled here (*e.g*., [31]), with the consequence that there is no buffering against perturbations in production or degradation of Ac-CoA. Thus, while changes in Ac-CoA at diauxic shift have been reported ([32], Fig 2A–B) and certainly have significant effects, such changes are controlled very tightly in the living cell.

The high positive LG associated with external serine transport is in accordance with experiments from our laboratory where this process was identified as the determinant for the control of sphingolipid flux and even more important than external palmitate input [33]. Again, the numerical values of the LG should not be taken at face value. Instead these LG results with respect to precursors should be interpreted on a scale of relative importance.

It might be interesting to note that the concentration of DHS-P is more strongly affected than that of PHS-P, while the opposite is true with respect to fluxes. Given the high LG, one could expect sphingoid base kinase and lyase to be more influential, but that does not seem to be the case.

#### 3.1.2. – Sphingolipid Block

Within the block of sphingolipid associated enzymes, SPT (*X*_57_) has the strongest effect (Figs. 3b,e). This effect is positive throughout and most clearly visible in the backbone sphingolipids and their phosphates. This finding is not surprising as SPT is commonly considered the first enzyme that controls entry into the sphingolipid pathway. Its crucial role has been widely documented [34,35]. As in the case of precursors, the metabolite and flux LG patterns with respect to DHS-P and PHS-P are opposite to each other.

Interestingly, the Elo1p (*X*_59_) complex exhibits negative LG for the sphingoid phosphates and backbones, indicating that increases tend to short-circuit production of sphingoid phosphates (or compete with it) and instead channels fatty acid precursors directly into ceramide, which is immediately (*i.e*., without sustained increase in concentration) used for IPC-g and the production of complex sphingolipids.

#### 3.1.3. – Glycerolipid Block

The LG of this block (Figs. 3c,f) are generally smaller in magnitude. G3P acyltransferase (*X*_49_) tends to have negative LG values because increases in this enzyme divert its substrate, palmitoyl-CoA (*X*_12_), away from sphingolipid metabolism and toward the glycerolipid pathway. The strongest effects are again seen in the sphingoid phosphates.

Interestingly, the LG associated with inositol-1-phosphate synthase (*X*_46_) are relatively high. The reasons are not intuitively evident, and we will explore in the laboratory whether this enzyme might be a regulator of the sphingolipids pathway. The fluxes through the IPC-g require phosphatidylinositol (PI), so that increases in enzymes that generate PI might significantly affect the production of complex sphingolipids, while enzymes that use PI in other processes, like PI kinase, have the opposite effect.

### 3.2. – Metabolic Consequences of Changes in Gene Expression

**Figure 4:**
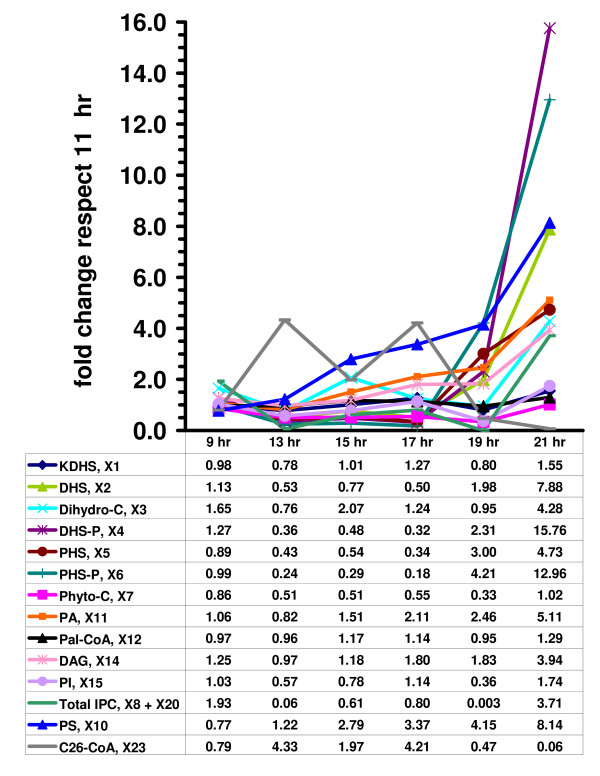
Selected fold changes in steady-state metabolite levels, according to the mathematical model, normalized with respect to metabolite values at 11 hrs (deduced from mRNA profiles in [1]).

Table 1 and Fig. 4 exhibit fold changes in metabolites that were calculated to result from changing enzyme activities according to DeRisi's microarray measurements. Where available, specific activities were adjusted according to reported values in the literature. For example, even though its mRNA changes at later times, the specific activity of PI synthase is known to remain within a range of 10–20% around its normal value, independent of external conditions ([36]: Fig. 2B and Table 2). It is to be expected that such discrepancies are due to post-transcriptional modifications. For published information of this type, we therefore decided to override the microarray results. For instance, we modified PI synthase levels only within a 10% range of its 11-hr value. At the same time, Homann [36] reported values for CDP-DG synthase (*X*_40_) and PS synthase (*X*_38_) that are consistent with the mRNA fold changes in the microarrays and therefore did not require adjustments.

Likewise, Table 4 presents fold changes in metabolites computed from simulated enzyme variations, according to the diauxic shift microarray time series [2]. These results are similar to those presented in Table 1 obtained under similar conditions with DeRisi's data. In fact, the correlation coefficient between Tables 3 and 4 (*r *= 0.88) is very similar to the value reported for replicate experiments in DeRisi's complete dataset (*r *= 0.87) [1]. To confirm the similarity between Tables 3 and 4 further, we performed a two-tailed Wilcoxon signed rank test, which detected no significant difference between the corresponding 22 dependent variables from both tables (*p *= 0.93). All of the above confirms that the two datasets are consistent and that the model implementation is reproducible.

**Table 4:** Steady-state-metabolite levels

			**FOLD CHANGE (normalized against 9.5 hr)**						
			
**Abbreviation**	**Symbol**	**Value (mol%)**	**0 hr**	**9.5 hr**	**11.5 hr**	**13.5 hr**	**15.5 hr**	**18.5 hr**	**20.5 hr**
KDHS	*X*_1_	0.005	1	1	0.79	1.01	1.28	0.80	1.57
DHS	*X*_2_	0.01	1.09	1	0.58	0.56	0.65	5.08	11.00
Dihydro-C	*X*_3_	0.036	1.67	1	0.75	2.07	1.20	0.72	4.06
DHS-P	*X*_4_	0.001	1.23	1	0.41	0.35	0.42	5.98	22.10
PHS	*X*_5_	0.05	0.84	1	0.51	0.33	0.53	6.76	5.94
PHS-P	*X*_6_	0.005	0.93	1	0.31	0.16	0.29	10.67	16.86
Phyto-C	*X*_7_	0.052	0.87	1	0.50	0.51	0.54	0.24	0.94
IPC-g	*X*_8_	0.102	2.03	1	0.05	0.58	0.76	0.002	3.41
CDP-DAG	*X*_9_	5.4	1.19	1	0.45	0.70	0.97	0.77	3.18
PS	*X*_10_	8.4	0.78	1	1.26	2.73	3.45	4.08	8.25
PA	*X*_11_	3	1.08	1	0.82	1.48	2.14	2.46	5.16
Pal-CoA	*X*_12_	0.01 ^(*)^	0.99	1	0.96	1.17	1.14	0.94	1.30
Serine	*X*_13_	2600 ^(*)^	1	1	1	1	1	1	1
DAG	*X*_14_	0.1	1.27	1	0.97	1.20	1.78	1.68	3.86
PI	*X*_15_	16.7	1.05	1	0.58	0.76	1.16	0.36	1.77
Inositol	*X*_16_	24.1 ^(*)^	1	1	1	1	1	1	1
CDP-Eth	*X*_17_	22	0.48	1	0.04	0.02	0.03	12.72	24.37
MIPC-g	*X*_18_	0.14	1.58	1	0.36	1.23	1.09	0.05	1.83
M(IP)2C-g	*X*_19_	0.0085	1.53	1	0.25	0.74	1.44	0.04	3.26
IPC-m	*X*_20_	0.918	2.03	1	0.05	0.58	0.76	0.002	3.41
MIPC-m	*X*_21_	1.26	1.58	1	0.36	1.23	1.09	0.05	1.83
M(IP)2C-m	*X*_22_	0.0765	1.53	1	0.25	0.74	1.44	0.04	3.26
C26-CoA	*X*_23_	0.5	0.87	1	3.60	3.62	2.57	0.07	0.03
Mal-CoA	*X*_24_	183 ^(*)^	1.03	1	0.35	0.47	0.31	0.84	10.52
Ac-CoA	*X*_25_	870 ^(*)^	1	1	1	1	1	1	1
Total IPC	*X*_8 _+ *X*_20_	1.02	2.03	1	0.05	0.58	0.76	0.002	3.41
Total MIPC	*X*_18 _+ *X*_21_	1.4	1.58	1	0.36	1.23	1.09	0.05	1.83
Total MIP_2_C	*X*_19 _+ *X*_22_	0.085	1.53	1	0.25	0.74	1.44	0.04	3.26
Total_Ceramide	*X*_3 _+ *X*_7_	0.088	1.20	1	0.60	1.15	0.81	0.44	2.21

(*) μM. Steady-state metabolite levels corresponding to published microarray results from the diauxic shift time course of Gash and Spellman [2]. Each case is represented as fold change of the value presented in Alvarez-Vasquez *et al*. [9]. "Zero" corresponds to quiescence.

### 3.3. – Sphingolipid Metabolism before and after the Diauxic Shift

**Figure 5:**
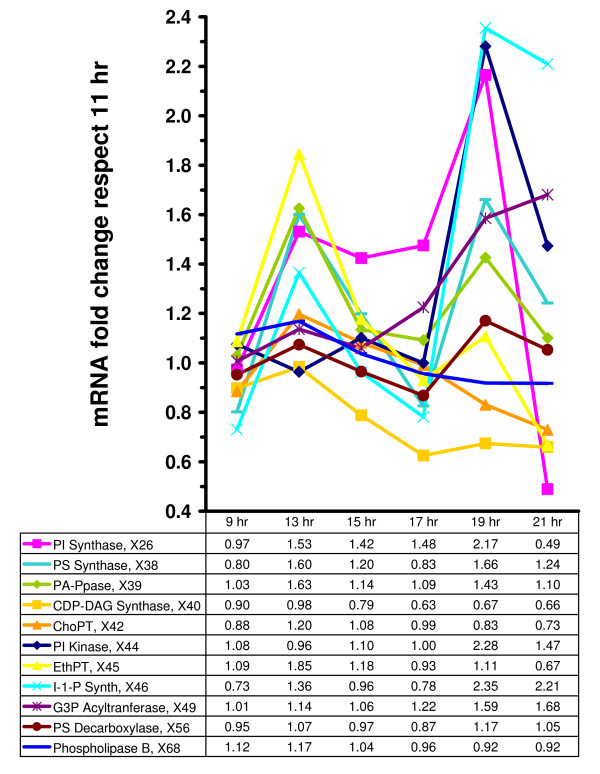
Fold change variation in mRNA levels at 9, 13, 15, 17, 19, and 21 hrs, normalized with respect to values during the exponential growth phase (11 hrs) [1]. Glycerolipid block.

**Figure 6:**
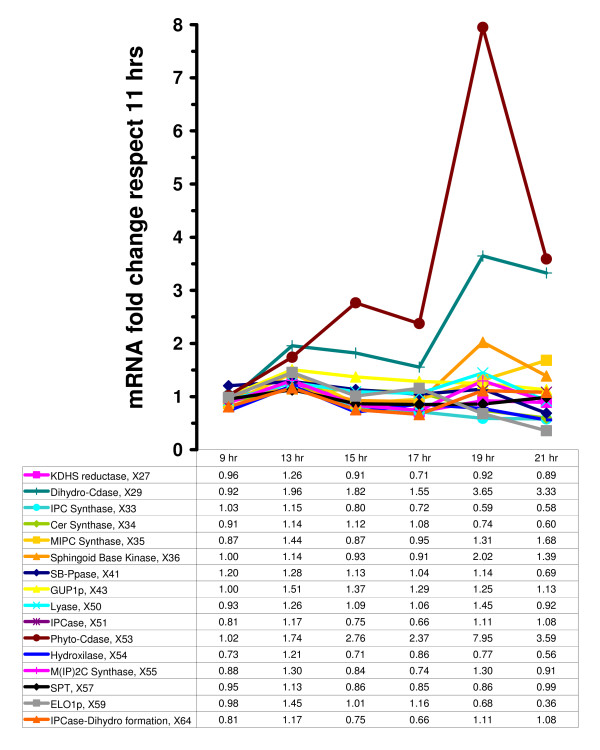
Fold change variation in mRNA levels at 9, 13, 15, 17, 19, and 21 hrs, normalized with respect to values during the exponential growth phase (11 hrs) [1]. Sphingolipid block.

**Figure 7:**
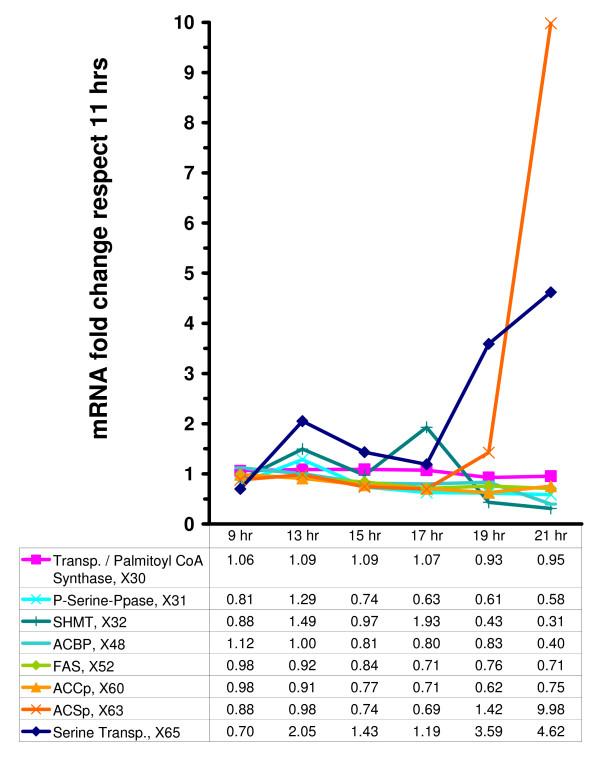
Fold change variation in mRNA levels at 9, 13, 15, 17, 19, and 21 hrs, normalized with respect to values during the exponential growth phase (11 hrs) [1]. Precursor block.

DeRisi's microarray results, as they pertain to variables in the model, are shown in Figs. 5, 6, and 7. They suggest that there are two relatively distinct phases, namely from the beginning of the experiment to time 17 hr (Phase 1, where glucose is available in relatively high concentrations), and time points 19 and 21 (Phase 2, where glucose is essentially depleted) (see Fig. 2). During Phase 1, the variables in the precursor block (Fig. 7) remain more or less close to the baseline level, *i.e*., within a factor of 2, which used to be considered the signal-to-noise threshold at the time when DeRisi's data were obtained. During Phase 2, ACSp and serine transport spike to 10- and 5-fold levels, respectively. The situation is similar for the glycerolipid and sphingolipid blocks (Figs. 5 and 6). During Phase 1, essentially all sphingolipid enzymes remain within a two-fold range, with the exception of phyto-ceramidase and dihydro-ceramidase, which moderately increase during the first phase and spike in Phase 2. Similarly in the glycerolipid block, the variables in the precursor block remain more or less close to the baseline level during Phase 1. During Phase 2, three variables exceed the signal-to-noise threshold with essentially the same fold increase of 2.3. They are I-1-P synthase, PI synthase, and PI kinase. It seems more than coincidence that these three out of eleven enzymes are directly associated with the dynamics of phosphatidyl inositol, the former two with its synthesis, and the latter with its further metabolism.

Microarray results on the sphingolipid pathway, taken by themselves, are difficult to interpret. However, application of the current dynamic model facilitates an additional type of exploration. Under the assumption that most regulation is transcriptional, it is easy to adjust enzyme activities in accordance with changes in mRNA levels and to compute the corresponding metabolic profiles by letting the model reach steady state. The results of this analysis are shown in Fig. 4. In Phase 1, when glucose is relatively plentiful, the only metabolites that are significantly increased over baseline (at 11 hr) are C_26_-CoA and Phosphatidylserine (PS). By and large, all other metabolites are at or below the 11-hr baseline level. Between 17 and 19 hrs, some dramatic changes take place. Most prominent are several-fold increases in the sphingoid phosphates DHS-P and PHS-P. DHS, PHS, PA, and DAG are also increased. Interestingly, C_26_-CoA becomes significantly depleted, while IPC increases four-fold.

Considering Figs. 4, 5, 6 and 7 simultaneously allows us to piece together the dynamics of sphingolipid metabolism during the diauxic shift. During Phase 1, C_26_-CoA is produced faster than it is utilized. This is not accomplished with one or two high-gain enzymes that are strongly up- or down-regulated, but through the coordinated regulation of many enzyme activities that are slightly increased or decreased. Initially Elo1p is up-regulated, but quickly returns to its original level, so that it alone cannot be responsible for the increase in C_26_-CoA levels. Notably, all backbone sphingolipids and sphingoid phosphates are reduced in concentration, which through the lyase reaction may lead to recycled palmitoyl-CoA, which is subsequently converted into C_26_-CoA. One could surmise that some important factor might be missing from the model, but even if that was the case, it would yield no explanation, because the mathematical model generates these higher levels of C_26_-CoA without additional input or manipulation. Further analysis of Figs. 4 and 5 suggests that sphingolipids are also utilized for remodeling, via GPI anchor enzymes as the Gup1p (*e.g*. [37]).

Of particular note is that serine transport is up-regulated. At least part of this increase of serine seems to be utilized for the generation of PS as PS synthase is up-regulated, along with PI synthase. On the other hand, and although the SPT level/activity is unchanged, a recent study from our group showed that increased serine influx drives flux through the sphingolipid pathway [33]. These results are in direct accordance with our LG analysis that revealed serine transport with the second highest value after the *de novo *palmitoyl-CoA production (Figs. 3a,d). It is also possible that PI is used for the creation of IPC, which however is immediately converted into complex sphingolipids. This connection is indirectly supported by earlier model simulations, in which an external inositol bolus led to decreases in ceramide levels and increases in the complex sphingolipids [9].

During Phase 2, drastic changes occur. Both ACSp and serine transport are strongly up-regulated, along with enzymes that shift production to sphingoid phosphates (Fig. 6). These may function as signaling agents that regulate/affect the genomic response to substrate depletion. Of note is that the shift toward sphingoid phosphates is not accomplished through up-regulation of the high-gain enzyme SPT (see Fig. 3b), which is often seen as the rate determining step of sphingolipid metabolism. Instead, low-gain ceramidases are increased in activity, along with lesser increases in sphingoid base kinase, while ceramide synthase is decreased. Thus, the coordinated action of these enzymes explains the increase in the sphingoid base phosphates. Also, Elo1p is reduced, which causes the formerly high C_26_-CoA levels to be used up, and IPC jumps from low amounts to four-fold over baseline. According to the gain profile, the loss in Elo1p activity also further increases the sphingoid phosphate levels. Concomitant with the jump in IPC, MIPC synthase and M(IP)_2_C synthase are also up-regulated, suggesting utilization of remaining sphingolipids in membrane material.

Overall, the cell seems to use a mixture of high-gain variables, including serine transport (with positive LG) and SHMT (with negative LG), and low-gain variables, such as ceramidases, to mount the sphingolipid responses to glucose depletion. ACSp, which has a very strong negative gain on sphingoid phosphates, is strongly up-regulated in Phase 2. One can only speculate that the effects of this up-regulation may be compensated by a lack of substrates for this reaction.

### 3.4. Validation Experiments

In a *de novo *experiment, subsequent to the model predictions, wild type sphingolipid levels were determined after 6 and 24 hours of growth, representing two clearly defined pre- and post- diauxic time points (Table 5). The fold-change results (last column of Table 5) show general qualitative concordance with the simulations from Tables 3 and 4 at 21 and 20.5 hr, respectively, and the predicted increase in most lipid levels after the diauxic shift corresponds with the experimental data.

**Table 5:** Experimental data before and after the diauxic shift

		[pmole/total sample]/total lipid phosphate				
		6 hr		24 hr		Fold change 24 hr/6 hr
				
		A	B	A	B	
				
OD 600 nm		0.34	0.35	2.31	2.19	
DHS	***X***_2_	0.45	0.40	1.44	1.49	3.44
Dihydro-C	***X***_3_	0.06	0.05	0.14	0.14	2.64
DHS-P	***X***_4_	0.04	0.04	0.06	0.05	1.24
PHS	***X***_5_	0.90	0.71	2.32	1.78	2.55
PHS-P	***X***_6_	0.02	0.02	0.37	0.30	17.83
Phyto-C	***X***_7_	7.80	7.38	9.20	8.05	1.14

Sphingolipid metabolite levels for wild type strain BY4742 at 6 and 24 hours. The right hand column shows the average fold change for each hour. A and B correspond to two independent experiments. See the material and methods secction for details.

There is a large quantitative difference for DHS (*X*_2_) and especially DHS-P (*X*_4_) in the experimental data vs. the major increases shown for those metabolites in the simulations. This disparity may result from the difference in strains and growth media in our experiments and those of DeRisi *et al*. [1] and Gasch *et al*. [2]. Another possibility is the need to include in the model the exogenous trafficking of sphingoid bases that potentially contribute to their levels, as well as the levels of the sphingoid base phosphates (*cf*. [27,38-41]).

## 4. – Discussion

The results from this study advance the development, analysis, and utility of a previous mathematical model for sphingolipid metabolism in yeast. The model was further refined with minor modifications encompassing the inclusion of enzymes for PI degradation, serine import, and GPI remodeling, and adjustments to ceramide synthase fluxes based on experimental data (see Appendix). LG analysis was conducted, and this provided further insight into the model and into the structure of the sphingolipid pathway in yeast. Finally, the model was applied to data extracted from the literature on changes in enzymes of sphingolipid metabolism, and this allowed for specific and novel insights into sphingolipid metabolism in during the diauxic shift. This use of a mathematical model, coupled with the integration of data from several sources, promises to have applicability beyond the system discussed here.

### 4.1. – Log Gains

LG analysis revealed several clusters of high LG. These were not randomly distributed throughout the pathway but showed very distinct patterns that allowed specific diagnostics. In general, the flux and metabolite LG analyses suggest that enzymes and metabolites of *de novo *fatty acid synthesis (Elo1p, acetyl-CoA carboxylase, CoA, acetate, acetyl-CoA synthetase), ATP, and the serine hydroxymethyl transferase have the strongest effect on the sphingolipid backbone metabolites (Fig. 3).

The second block consisted of relatively high gains that are presumably real, demonstrating that the sphingolipid signaling pathway has the capacity to amplify specific inputs. This cluster of high-gain dependent variables consists of dihydrosphingosine, dihydrosphingosine 1-phosphate, phytosphingosine, phytosphingosine 1-phosphate and inositol phosphorylceramide, which show moderate to strong responses to changes in a number of enzyme activities (Fig. 3).

Other high gains appeared to be modeling artifacts. These were associated with acetyl-CoA, whose gains in some cases reached up to 200 (Fig. 3a), as well as the model inputs, serine, glucose 6-phosphate, cytidine-diphosphate ethanolamine (CDP-Eth) and inositol (log gains of the last three are not shown) which exhibited less dramatic gains that nonetheless were much higher than one would normally expect in a metabolic pathway. In fact, the metabolites associated with these gains are very prevalent and involved in numerous pathways. However, in the model they appear only as part of the model input, and their dynamics is controlled entirely by a handful of variables, which in the global picture of an entire cell constitute but one aspect of their metabolism (see Fig. 1). While the gains suggest that these variables at the "fringes" of the model are not adequately represented, the initial model was designed to capture the dynamics of sphingolipids, and keeping the input variables relatively close to their baseline levels, the observed responses in other variables were not unreasonably affected by the high gains. Nonetheless, because fatty acids and serine play critical roles for the dynamics of sphingolipids [33,34], we found it useful to expand the original model by including external serine, specific components of fatty acid synthesis, phospholipase B, and the remodeling enzyme Gup1p that, together with the sphingosine-phosphate lyase reaction, are the two ultimate exit routes out of the system, and to revisit sensitivity and gain profiles characterizing crucial nodes in the model.

Taken together, coupling established LG analysis with biochemical insights begins to define at least one subset of parameters that stand out as being of potential significance. This type of analysis should aid the experimentalist in focusing on these more significant pathway components (enzymes and/or metabolites) for further study.

### 4.2. – Changes During the Diauxic Shift: a paradigm for pathway analysis through mining databases and applying mathematical analysis

Several key features emerged from the calculated changes in sphingolipids during the diauxic shift.

First, significant increases were predicted in complex sphingolipids. The diauxic shift demands increases in membrane lipids, which may be explained in part by a sudden increase in density of immature daughter yeast cells occurring during the late exponential growing phase [42]. The increases in IPC, MIPC, and MIP_2_C in the post diauxic phase are consistent with this requirement (Table 1 and Fig. 4).

A possible explanation for the drastic decrease in complex sphingolipids seen at 19 hr is the decrease in the levels of PI (Table 1 and Fig. 4). This close link between complex sphingolipids and PI is expected based on the effect that inositol and PI have on ceramide levels [9].

Second, significant increases were observed in LCB's and LCBP's at 19 and 21 hrs (Tables 1, 4 and Fig. 4), which reflect the integrated response of a group of synergistically functioning enzymes and suggest functional importance for these lipids. These model results are in close agreement with the published experimental results of Lanterman and Saba [43] who observed a sharp increase in sphingoid base 1-phosphates during the diauxic shift. Moreover, an increase in sphingosine kinase (Fig. 6) at the diauxic shift was reported by Dickson and co-workers [27] who also suggested that its products PHSP and DHSP may have a physiological role in the diauxic shift or the stationary phase that follows this cellular event.

*Serine Palmitoyl Transferase*. Even though the *LCB *1/2 mRNA levels increase at the diauxic shift by only 20% with respect to their 11 hr values, the *K*_*M*_'s of SPT with respect to serine and palmitoyl-CoA are close to the cell levels of these metabolites, and therefore the concentrations of the substrates exert an important effect on the overall flux throughout the sphingolipid pathway.

The increases in ceramidase at later times contribute significantly to the sphingoid backbones and sphingoid phosphates at the expense of the levels of ceramide. By contrast, the three *ELO*'s mRNA levels decreased at later times [1]. In the model these three mRNA's were considered collectively and weighted by decreasing the Elo1p (*X*_59_) by a 32% and 64% at 19 and 21 hrs respectively. This decrease exerted great influence on the metabolomic profile because Elo1p shows one of the highest LGs reported (Fig. 1 and Fig. 3b). The concomitant decrease of the three *ELO*'s suggests a coordinate regulation of the long and very long chain ceramides.

Because the IPCase activity in the model increased only slightly right after the diauxic shift (Fig. 6), it is probable that the observed increase in dihydroceramide at 21 hr (Table 1 and Fig. 4) is mainly caused by the *de novo *pathway. Indeed, when one compares the phytoceramide levels at 9 and 21 hrs, one finds an increase of 20% (Fig. 4), which is in line with observations of Vaena de Avalos *et al*. ([44]: Fig, 5), who showed that phytoceramide increases about 40% in wild type cells between early-log phase and after the diauxic shift.

In accordance with the mRNA levels of *DPL1 *pre and post diauxic shift, the breakdown of sphingoid phosphates through lyase (*X*_50_) increased 45% at 19 hr and returned to the basal values at 21 hr (Fig. 6). This creates an effective bottleneck for the LCBPs formed at later times and contributes to the accumulation of LCBs and LCBPs (Table 1 and Fig. 4).

*LCB4 *and *LCB3 *also contribute to the rise in LCBPs at the expense of the LCB's. *LCB4*, coding for one of the two sphingosine base kinases (*X*_36_), increased 100% at 19 hr. On the other hand, mRNA of *LCB3*, which codes for one of the sphingoid base phosphate phosphatases (*X*_41_) decreased 40% from pre to post diauxic shift.

Other notable changes are those in *RSB1*, a suggested ATP dependent flippase or transporter for LCBs which may catalyze LCB movement from the inner to the outer leaflet of the plasma membrane [40]. In the post diauxic phase, the mRNA level of *RSB1 *increased four fold with respect to its value at the early exponential phase, suggesting a requirement for increased plasma membrane LCBs translocation.

Svf1p was recently shown to be involved in the regulation of sphingoid bases and their phosphates [45]. Ypk1p is a kinase similarly involved in mediating the action of sphingoid bases [46]. Interestingly, both genes were recently associated with the regulation of the transient growth arrest observed at the diauxic shift [45]. Because we predict an increase in sphingoid bases at later times (Fig. 4) and because high levels of LCBs are toxic to wild type cells [47], the decrease in post diauxic mRNA levels for *SVF1 *and *YPK1 *to about 50% [1], could possibly serve to regulate metabolism and function of LCBs.

Third, there are many possible relationships between the changes in enzymes of sphingolipid metabolism and calcium homoestasis. According to Birchwood *et al*. [48], the accumulation of intracellular sphingoid base phosphates after the diauxic shift might serve as a regulator of calcium signaling. Analyzing the mRNA database for genes related to Ca^+2 ^influx, such as *BNI1*, *FAR1*, *MID1*, and *CRZ1 *[48,49], we noticed that these decrease at the diauxic shift. Also increases in the mRNA for *PMC1 *(a gene that codes for vacuolar Ca^2+^-ATPase) suggest the tendency to maintain a low cytoplasmatic Ca^2+ ^level at this cellular moment.

Fourth, the model predicts some notable changes in glycerolipids, which increase at later times. It is important to remark here that the glycerolipids represented in the model are restricted to the metabolites directly related with the sphingolipid pathway and that not all routes involved in glycerolipid metabolism are included. For example, the cardiolipin pathway is not included in the model. This route becomes important when the cell needs to increase the mitochondrial biomass after the diauxic shift. In fact the deletion of the phosphatidylglycerophosphate synthase, the first and rate limiting enzyme of the cardiolipin pathway, is not essential to cell viability but causes growth dependence on fermentable carbon sources because of mitochondrial dysfunction [50]. Pilot simulations (not shown) of variations in phosphatidylglycerophosphate synthase show only small effects on the global metabolic system.

Table 1 shows that, in general, the glycerolipid metabolites increase after the diauxic shift. Specifically, they drive the flux mainly through PS production at the expense of PI production [50]. At the diauxic shift, PA accumulation occurs in part due to a 40% decrease in CDP-DAG synthase activity with respect to its 11 hr value (Fig. 5). In light of the reported effects of inositol on ceramide levels [9] and on the glycerolipid pathway [51], we are led to suggest that this regulation is another important contribution to formation of complex sphingolipids, especially at later times (*e.g*., the 19 hr time point).

Table 5 shows a qualitative concordance between experimental data and the simulations at well defined cellular moments, which indicates that the simulations and microarray implementation are reasonable. The experimental data suggest future fine tuning of the model to include a route for trafficking of the sphingoid bases and their phosphates to and from the cell.

In conclusion, the computational study presented here suggests that the coordination of sphingolipid involvement in the diauxic shift in yeast is achieved through multiple small modifications of functional clusters of mRNAs rather than through large alterations in just a few "key" genes or metabolic steps. In most cases, the variations in mRNA expression are less than 2-fold, which implies that expression studies have to achieve a level of relatively high accuracy, if they are to detect subtle, coordinated control mechanisms.

The combined approaches of this study may also represent an important new paradigm in metabolomic studies whereby mathematical modeling of metabolic pathways is employed to mine and analyze gene expression studies. Given the rapidly increasing accumulation of microarray-based gene expression datasets, such an approach may result in very important insights into the changes, behavior, and possibly function of specific metabolic pathways.

## Authors' contributions

FAV developed the model, conducted the simulations and drafted the initial version of the manuscript. KJS performed the experiments. YAH and EOV conceived and supervised the collaboration and overall strategy of the project, and edited the manuscript.

## Appendix

### A.1 – Modifications of the published model

The model of Alvarez-Vasquez *et al*. [8,9] is based on the metabolic pathway structure shown in Fig. 1 and mathematically represented as a Generalized Mass Action (GMA) model within Biochemical Systems Theory (*e.g*., [52-54]). In this formulation, all chemical conversions, enzyme catalyzed reactions and transport processes are described as products of power-law functions that contain those and only those metabolites, enzymes and other factors that directly affect this process. The processes are collected to form a system of ordinary differential equations. The original model was slightly refined here to account for information that emerged as more important than was assumed in the design of the original model.

#### A.1.1

Enzyme Plb3p, which catalyzes the degradation of phosphatidylinositol (PI), was added to the collection of processes metabolizing PI. Merkel *et al*. [55] reported that deletion of this enzyme reduces PI breakdown by 50%.

#### A.1.2

Gup1p was included explicitly as a representative enzyme for GPI remodeling. In the original model, the independent variable responsible for driving the phytoceramide flux toward GPI remodeling (*X*_43_) was not associated with a specific enzyme. Recently, Bosson *et al*. [37] identified Gup1p as a key enzyme involved in the incorporation of PHS-C26:0 and PHS-C26:0-OH ceramide into the anchor for GPI remodeling.

#### A.1.3

Serine transport was explicitly incorporated. Research in our lab has identified this variable as very important in the control of flux through the sphingolipid pathway [33]. Many permeases are known to play a role in serine transport. We chose the polyamine permease AGP2p as representative of serine import because it plays an important role in amino acid transport [56], with mRNA levels changing significantly during the diauxic shift.

#### A.1.4

The quantification of ceramide synthase fluxes was fine-tuned. *i.e *experiments performed by the Lipidomics Core of our research group suggest that an external inositol bolus causes the concentration of phytoceramide to decrease more strongly than that of dihydroceramide (data not shown). This result was implemented as a numerical alteration of the former ceramide synthase fluxes in this model.

### A.2 – Definitions of Logarithmic Gains (LG)

To simplify the assessment of LG terms, the model was implemented as an S-system by aggregating all influxes and effluxes for each time-dependent variable into a single power-law term each (*e.g*., [52-54]). This procedure is legitimate, because gains and sensitivities are computed at the steady state, where the GMA and S-system models are equivalent. Beyond the steady state, we showed in Alvarez-Vasquez *et al*. [8] that alternative formulations of the sphingolipid pathway (including Michaelis-Menten, S-system, and GMA models) yield essentially equivalent responses.

Logarithmic gains come in two varieties. Each *metabolite LG *is defined as the ratio of the percent change in a dependent variable (typically a metabolite concentration) *X*_*i *_to the percent change in an independent variable (typically an input, enzyme activity or transport step) *X*_*k*_, while all other independent concentrations and parameters are held constant. It is thus defined as

L(Xi,Xk)=Lik=(∂XiXi/∂XkXk)0=(∂Xi∂Xk⋅XkXi)0=(∂(log⁡Xi)∂(log⁡Xk))0,
 MathType@MTEF@5@5@+=feaafiart1ev1aaatCvAUfKttLearuWrP9MDH5MBPbIqV92AaeXatLxBI9gBaebbnrfifHhDYfgasaacPC6xNi=xI8qiVKYPFjYdHaVhbbf9v8qqaqFr0xc9vqFj0dXdbba91qpepeI8k8fiI+fsY=rqGqVepae9pg0db9vqaiVgFr0xfr=xfr=xc9adbaqaaeGacaGaaiaabeqaaeqabiWaaaGcbaGaemitaWKaeiikaGIaemiwaG1aaSbaaSqaaiabdMgaPbqabaGccqGGSaalcqWGybawdaWgaaWcbaGaem4AaSgabeaakiabcMcaPiabg2da9iabdYeamnaaBaaaleaacqWGPbqAcqWGRbWAaeqaaOGaeyypa0ZaaeWaaeaadaWcaaqaaiabgkGi2kabdIfaynaaBaaaleaacqWGPbqAaeqaaaGcbaGaemiwaG1aaSbaaSqaaiabdMgaPbqabaaaaOGaei4la8YaaSaaaeaacqGHciITcqWGybawdaWgaaWcbaGaem4AaSgabeaaaOqaaiabdIfaynaaBaaaleaacqWGRbWAaeqaaaaaaOGaayjkaiaawMcaamaaBaaaleaacqaIWaamaeqaaOGaeyypa0ZaaeWaaeaadaWcaaqaaiabgkGi2kabdIfaynaaBaaaleaacqWGPbqAaeqaaaGcbaGaeyOaIyRaemiwaG1aaSbaaSqaaiabdUgaRbqabaaaaOGaeyyXIC9aaSaaaeaacqWGybawdaWgaaWcbaGaem4AaSgabeaaaOqaaiabdIfaynaaBaaaleaacqWGPbqAaeqaaaaaaOGaayjkaiaawMcaamaaBaaaleaacqaIWaamaeqaaOGaeyypa0ZaaeWaaeaadaWcaaqaaiabgkGi2kabcIcaOiGbcYgaSjabc+gaVjabcEgaNjabdIfaynaaBaaaleaacqWGPbqAaeqaaOGaeiykaKcabaGaeyOaIyRaeiikaGIagiiBaWMaei4Ba8Maei4zaCMaemiwaG1aaSbaaSqaaiabdUgaRbqabaGccqGGPaqkaaaacaGLOaGaayzkaaWaaSbaaSqaaiabicdaWaqabaGccqGGSaalaaa@79F8@

where the subscript 0 refers to the chosen operating point, which usually coincides with the steady state.

In an analogous fashion, a *flux LG *is defined as:

L(Vi,Xk)=LVik=(∂ViVi/∂XkXk)0=(∂Vi∂Xk⋅XkVi)0=(∂(log⁡Vi)∂(log⁡Xk))0
 MathType@MTEF@5@5@+=feaafiart1ev1aaatCvAUfKttLearuWrP9MDH5MBPbIqV92AaeXatLxBI9gBaebbnrfifHhDYfgasaacPC6xNi=xI8qiVKYPFjYdHaVhbbf9v8qqaqFr0xc9vqFj0dXdbba91qpepeI8k8fiI+fsY=rqGqVepae9pg0db9vqaiVgFr0xfr=xfr=xc9adbaqaaeGacaGaaiaabeqaaeqabiWaaaGcbaGaemitaWKaeiikaGIaemOvay1aaSbaaSqaaiabdMgaPbqabaGccqGGSaalcqWGybawdaWgaaWcbaGaem4AaSgabeaakiabcMcaPiabg2da9iabdYeamnaaBaaaleaacqWGwbGvdaWgaaadbaGaemyAaKgabeaaliabdUgaRbqabaGccqGH9aqpdaqadaqaamaalaaabaGaeyOaIyRaemOvay1aaSbaaSqaaiabdMgaPbqabaaakeaacqWGwbGvdaWgaaWcbaGaemyAaKgabeaaaaGccqGGVaWldaWcaaqaaiabgkGi2kabdIfaynaaBaaaleaacqWGRbWAaeqaaaGcbaGaemiwaG1aaSbaaSqaaiabdUgaRbqabaaaaaGccaGLOaGaayzkaaWaaSbaaSqaaiabicdaWaqabaGccqGH9aqpdaqadaqaamaalaaabaGaeyOaIyRaemOvay1aaSbaaSqaaiabdMgaPbqabaaakeaacqGHciITcqWGybawdaWgaaWcbaGaem4AaSgabeaaaaGccqGHflY1daWcaaqaaiabdIfaynaaBaaaleaacqWGRbWAaeqaaaGcbaGaemOvay1aaSbaaSqaaiabdMgaPbqabaaaaaGccaGLOaGaayzkaaWaaSbaaSqaaiabicdaWaqabaGccqGH9aqpdaqadaqaamaalaaabaGaeyOaIyRaeiikaGIagiiBaWMaei4Ba8Maei4zaCMaemOvay1aaSbaaSqaaiabdMgaPbqabaGccqGGPaqkaeaacqGHciITcqGGOaakcyGGSbaBcqGGVbWBcqGGNbWzcqWGybawdaWgaaWcbaGaem4AaSgabeaakiabcMcaPaaaaiaawIcacaGLPaaadaWgaaWcbaGaeGimaadabeaaaaa@7A63@

where *V*_*i *_represents a given (aggregated) flux, and *X*_*k *_and the subscript 0 are defined as before.

### A.3 – Assumptions Underlying the Translation of Gene Expression into Metabolic Changes

The conversion of gene expression profiles into changes in enzyme activities (*cf*. [5-7]) relies on the following assumptions.

#### A.3.1

The direct proportionality between a change in mRNA level and the corresponding enzymatic activity presupposes strictly transcriptional regulation, while posttranslational modifications are ignored. There is circumstantial evidence suggesting that this assumption is quite realistic in the case of heat stress responses in yeast.

#### A.3.2

It is assumed that the mathematical model [8,9] accurately describes sphingolipid metabolism at the 11 hr time point in DeRisi's dataset and the 9.5 hr time point in Gasch's dataset. These time points were chosen because they fall within the exponential growth phase, for which most of the data for our model design were obtained. It is similarly assumed that the model structure, with adjusted enzyme activities, captures the dynamics of sphingolipid metabolism with sufficient accuracy over the time span in DeRisi's and Gasch's datasets.

#### A.3.3

It is assumed that the values of internal serine (*X*_13_), inositol (*X*_16_), and acetyl-CoA (*X*_25_) do not change significantly between the time points of analysis. The rationale for this assumption is that these metabolites are involved in very many cellular processes that are not adequately represented in the model. Thus, while they are buffered in reality by a slew of metabolic processes, the model does not allow for such buffering, because peripheral reactions and pathways outside sphingolipid metabolism are not modeled. In fact, experimental results from our lab (A. Cowart, pers. comm.) suggest that internal serine is maintained close to its baseline concentration of 2.6 mM throughout the diauxic shift. The production of inositol depends directly on G-6-P through catalysis mediated by I-1-P synthase. However, G-6-P is not comprehensively modeled because, for instance, utilization for trehalose or pentose production is not included in the model. Because the branch point at G-6-P is highly regulated and we do not have specific information on changes in inositol concentrations *in vivo*, we consider inositol as constant by default.

Acetyl-CoA is increasingly notably at the diauxic shift [32] because it is required for the tricarboxilic acid and glyoxylate cycles and enhances respiration. However, like G-6-P, acetyl-CoA is involved in many processes that are not captured by the model, thereby causing inconsistencies in some simulations (not shown). Our default is therefore to keep this variable constant, assuming that the effective acetyl-CoA flux into the sphingolipid and glycerolipid pathways is tightly regulated within a narrow range; in fact FAS (*X*_52_) and ACCp (*X*_60_) mRNA's are down-regulated at the diauxic shift, thereby probably affecting the acetyl-CoA influx (Fig. 7).

#### A.3.4

The steady states obtained for each new mRNA profile were obtained with a model where all fluxes are balanced, while the previous model contained some unbalanced fluxes among the complex sphingolipids, reflecting the need for membrane in the exponentially growing culture [9].
